# Prevalence of HIV-1 pretreatment drug resistance among treatment naïve pregnant women in Bissau, Guinea Bissau

**DOI:** 10.1371/journal.pone.0206406

**Published:** 2018-10-31

**Authors:** Sten Wilhelmson, Fredrik Månsson, Jacob Lopatko Lindman, Ansu Biai, Joakim Esbjörnsson, Hans Norrgren, Marianne Jansson, Patrik Medstrand, the SWEGUB CORE group

**Affiliations:** 1 The Department of Translational Medicine, Lund University, Malmö, Sweden; 2 The Department of Clinical Sciences Lund, Lund University, Lund, Sweden; 3 The National Public Health Laboratory, Bissau, Guinea-Bissau; 4 The Department of Laboratory Medicine, Lund University, Lund, Sweden; University of Cincinnati College of Medicine, UNITED STATES

## Abstract

**Background:**

With increased access to antiretroviral treatment (ART) in sub-Saharan Africa emergence of HIV-1 pretreatment drug resistance constitutes a serious risk. This may lead to rapid virological failure in subjects initiating ART, and mother-to-child transmission despite prophylaxis.

**Methods:**

Treatment-naïve pregnant women from four antenatal care clinics in Bissau, Guinea-Bissau, were enrolled from October 2016 to November 2017. Genotypic resistance testing and phylogenetic subtype analysis was performed on 48 specimens.

**Results:**

Forty eight women met the survey inclusion criteria. All specimens were successfully amplified and genotyped. Specimens from five women were associated with HIV-1 drug resistance mutations. Four carried mutations exclusively linked to non-nucleoside reverse transcriptase inhibitors (NNRTIs) (*K103N*, *K103N/S*) and one carried mutations to both NNRTIs (*G190S*, *K101E*) and nucleoside reverse transcriptase inhibitors (NRTIs) (*M184V*). These results corresponded to 10.4% (95% CI: 4.5–22.2%), 2.1% (95% CI: 0.4–10.9%) and 0% (95% CI: 0.0–7.4%) drug resistance mutations to NNRTIs, NRTIs and protease inhibitors, respectively. HIV-1 circulating recombinant form *02AG* was most commonly found, followed by HIV-1 *sub-subtype A3*. Subtype/CRF was not associated with drug resistance mutations.

**Conclusion:**

Our study reports a 10.4% prevalence of pretreatment drug resistance to NNRTIs in HIV-1-infected pregnant women in the capital Bissau, Guinea Bissau. Since NNRTIs are part of first-line ART in the country, baseline resistance screenings or adjustment of national treatment guidelines should be considered as antiretroviral treatment programs are scaled up.

## Introduction

Guinea-Bissau is a small multi-ethnic West African country with 1.8 million inhabitants, of whom 300,000 reside in the capital, Bissau [1]. The country ranks amongst the poorest in the world [1]. The human immunodeficiency virus type 1 (HIV-1) prevalence among adults, 15–49 years, is estimated to 3.1%, but is higher among women than men (3.8% vs. 2.4%) [2]. The national antiretroviral treatment (ART) program in Guinea-Bissau was initiated in 2005. As in most low and middle income countries (LMIC), ART follows the WHO guidelines with use of two nucleoside reverse transcriptase inhibitors (NRTIs) and one non-nucleotide reverse transcriptase inhibitor (NNRTI) [3]. About 35% of eligible HIV positive adults and 15% of the children received ART in 2016, while the proportion of pregnant women who received prevention from mother to child transmission (PMTCT) prophylaxis was 85% [2]. Furthermore, high numbers of loss to follow-up, mortality and development of HIV drug resistance (DR) during treatment (acquired DR) have been reported in the country [4].

The emergence of HIV-1 DR constitutes a threat to ART programs in resource-limited settings. Various factors drive the emergence of HIV-1 DR, including poor adherence and logistical barriers [5], high replication and mutation rates of HIV-1, and low genetic barrier for development of resistance to standardized first-line regimes [6]. Acquired DR as well as DR in treatment-naïve people (pretreatment DR) have increased in LMIC over the last decade [7, 8]. From a public health perspective it is important to monitor levels of acquired and pretreatment DR in order to forecast long term success of ART programs [9]. On an individual level pretreatment DR is of particular importance in LMIC, where virological monitoring and drug resistance screening is normally not part of patient management. Thus, individuals infected with drug resistant HIV-1 strains are at risk of not benefitting from the treatment, which may lead to disease progression and onward transmission.

Various HIV subtypes and circulating recombinant forms (CRF) cause infection in humans. Recombination between HIV subtypes and CRFs have been estimated to account for approximately 20% of the global HIV infections [10]. *CRF02_AG* is the most prevalent variant in several West African countries and accounts for 39–83% of the infections in this area [11–13]. Three major HIV subtypes/CRFs have been described in Guinea-Bissau: *CRF02_AG*, *sub-subtype A3*, and recombinants of *CRF02_AG* and *A3* referred to as *A3/02* [12, 13]. This may be important in the HIV epidemic since different viral variants have been linked to differences in viral load [14–16], disease progression rate [12], vertical transmission rate [17] and propensity to develop resistance to ART [18].

Documenting the profile of DR in HIV-1-infected pregnant women is crucial for improving the efficacy of maternal ART and prophylaxis in infants, and is also a preferred approach to estimate pretreatment DR (19). This can also help policy makers in the process of designing future national HIV treatment guidelines. Thus, in the context of increasing prevalence of acquired DR, and to gain an understanding of the effectiveness of contemporary ART in Guinea-Bissau, the aim of the current study was to estimate the level of pretreatment DR among pregnant women in the country. Moreover, since resistance data linked to information regarding HIV-1 subtypes and recombinants circulating among pregnant women have not been reported in Guinea-Bissau previously, this was also studied.

## Methods

### Study design and participants

Pregnant women who tested positive for HIV-1 in antenatal screening at four antenatal care clinics in the capital Bissau: Bairro Militar Health Centre, Antula Health Centre, Quelele Health Centre and Plack-II Health Centre, were asked for participation in the study. All participants finalized a questionnaire regarding previous HIV testing, antiretroviral treatment/mother-to-child prophylaxis as well as questions of a socio-economical nature. The survey aimed to follow the World Health Organization (WHO) recommended threshold survey methodology [19]. However, the WHO threshold survey methodology was under revision at the time of this study, and hence, we used the pre-revised guidelines. Original inclusion criteria were laboratory confirmation of HIV infection, age <25 years and no previous pregnancies. Due to frequent stock outs of HIV tests and a slower inclusion rate as a result, and in order not to prolong the study period, we omitted the age limit of 25 years and also included women with previous pregnancies. A total of 52 reportedly antiretroviral-naïve HIV-infected pregnant women were enrolled from October 2016 to November 2017. All participants that tested HIV positive were counselled and informed about antiretroviral treatment (ART), and were offered ART through the local health centre or at centralised services within Bissau City.

### Sample management

Determine (Abbott Diagnostic Division, Hoofddorp, Holland) was used for pretreatment HIV diagnosis at the antenatal care clinics. Samples were transported to the laboratory for national health (LNSP) and analyzed for CD4 absolute count, CD4% and haemoglobin count using FACSPresto—Near Patient CD4 counter (Becton Dickinson, NYSE:BDX, USA). A confirmatory HIV-1 discriminatory test was performed using Geenius HIV 1/2 confirmatory assay (Bio-RAD). Plasma was separated from whole blood by centrifugation and stored at -20 C until transported on dry ice for further storing in -80 C and genotyping at the Clinical Virology section at Lund University, Sweden.

### Drug resistance genotyping

RNA was extracted from plasma using the QIAamp Viral RNA Mini Kit (Qiagen). Reverse transcription and PCR amplification of HIV-1 *pol* gene were done using One-Step SuperScript III RT/Platinum Taq High Fidelity Enzyme Mix (ThermoFisher Scientific), using JA269 and JA272 primers [20]. For nested PCR, High Fidelity Platinum Taq DNA Polymerase, (ThermoFisher Scientific) was used, with primers JA270 and JA271 [20], resulting in a PCR fragment of 1086 bases. The PCR products were sequenced in both directions with six primers described by Zhou et al. [21] using the BigDye terminator kit v 1.1 (Applied Biosystem) followed by sequence analysis on an ABI PRISM 3130 ×l genetic analyzer (Applied Biosystem). Sequence assembly and editing were performed using RECall V 2.0 HIV sequencing analysis tool [22]. The final length of all the sequences following removal of regions corresponding to the primers, editing and alignment was 1035 bases, corresponding to nucleotide positions 2268–3302 of HXB2 (GenBank accession number K03455). Sequence quality control was performed using the quality control program of the Los Alamos HIV sequence database [23]. The presence of drug resistance mutations (DRM) was assessed using the Stanford Genotypic Resistance Interpretation Algorithm [24]. DRM were examined according to the calibrated population resistance (CPR) tool v6.0 beta [25] (http://cpr.stanford.edu/cpr/servlet/CPR), based on the WHO surveillance transmitted drug resistance mutation list of 2009 [26]. Pretreatment DR was referred to as low (< 5%), moderate (5–15%), or high (>15%) [27].

### HIV subtyping

All sequences were screened for recombination using RDP v.3.44 [28]. The sequences were subtyped through phylogenetic analysis with group M HIV reference sequences from Los Alamos HIV database [23] with the addition of *sub-subtype A3* polymerase sequences [29]. Phylogenetic subtyping was verified using COMET and jpHMM [30, 31]. Sequences were aligned using ClustalX2 and then edited to a final length of 1035 bases for each sequence using BioEdit [32, 33]. A Maximum likelihood phylogenetic tree was constructed using the online version of PhyML with the GTR+I+Γ nucleotide substitution model (using estimated proportion of invariable sites and four gamma categories) and SPR to estimate the tree topology. Branch support was determined with aLRT-SH (approximate likelihood ratio test Shimodaira-Hasegawa like) implemented in PhyML. A branch in the phylogeny with an aLRT-SH value ≥0.9 was considered significant [34, 35]. Cluster analysis was performed using ClusterPicker, where phylogenetic linkage was defined as an aLRT-SH branchsupport ≥0.9 and a genetic distance threshold of 5% [36].

### Statistical analysis

Statistical tests were performed using SPSS 24 (IBM Corp., Armonk, NY, USA). Resistance prevalence analyses were performed on 48 patients. Variables are expressed as medians with interquartile ranges (IQRs). Level of DR prevalence was determined with a confidence interval (CI) of 95% using the Wilson method. Univariate analysis for association between resistance mutations and sociodemographic/laboratorial variables was done using 2-tailed Fisher’s exact test.

## Ethical approval

The study was approved by the ethical committees of the National Health Ethics Committee in Guinea-Bissau (Ref 038/CNES/INASA/2016) and the Regional Ethical Review Board, Lund University, Sweden (Dnr 2016/426). All participants received information about the study before inclusion, and provided oral and written informed consent. To ensure confidentiality, all study data was managed under code.

### Availability of data

Nucleotide sequences reported in this study have been deposited in the Genbank repository (Accession Numbers: MH605452-MH605505).

## Results

### Study population

Fifty-two pregnant women met the survey inclusion criteria. Among them, 48 were confirmed HIV-1 infected and four were confirmed HIV-2 infected. The HIV-1 pol gene was successfully amplified and sequenced from plasma RNA of all 48 HIV-1 infected individuals. Among the 48 confirmed HIV-1 positive participants, 21 (43.8%) were enrolled at Bairro Militar Health Centre, nine (18.8%) at Antula Health Centre, ten (20.8%) at Quelele Health Centre and eight (16.7%) at Plack-II Health Centre, all located in the capital Bissau. Demographic characteristics of participants are shown in Table 1. Data in regard to absolute CD4 count, CD4%, age and previous pregnancies were missing for two, four, four and three of the women, respectively. The median CD4^+^ T-cell count was 415 (IQR: 292–562) cells/mL and the median CD4^+^ T-cell percentage (CD4%) was 19.4 (IQR: 16.1–25.9). The median age was 25 (IQR: 22–28) years and the number of women with previous pregnancies was 22/45 (48.9%).

**Table 1:** Demographic characteristics of enrolled participants (n = 48).

Characteristic	Value^1^
**Median Age, years (IQR)**	25 (22–28)
**Earlier pregnancies**	
Yes	22 (45.8)
No	23 (47.9)
N/A	3 (6.3)
**Education, years in school**	
No education	15 (31.3)
1–8	11 (22.9)
9–12	10 (20.8)
N/A	12 (25)
**Marital Status**	
Married	32 (66.7)
Not Married	10 (20.8)
N/A	6 (12.5)
**Ethnic group**	
Fula	11 (22.9)
Mandinga	11 (22.9)
Balanta	9 (18.8)
Others	11 (22.9)
N/A	6 (12.5)

^1^ Values are n (%) except where otherwise indicated

### Subtype distribution

**Fig 1:**
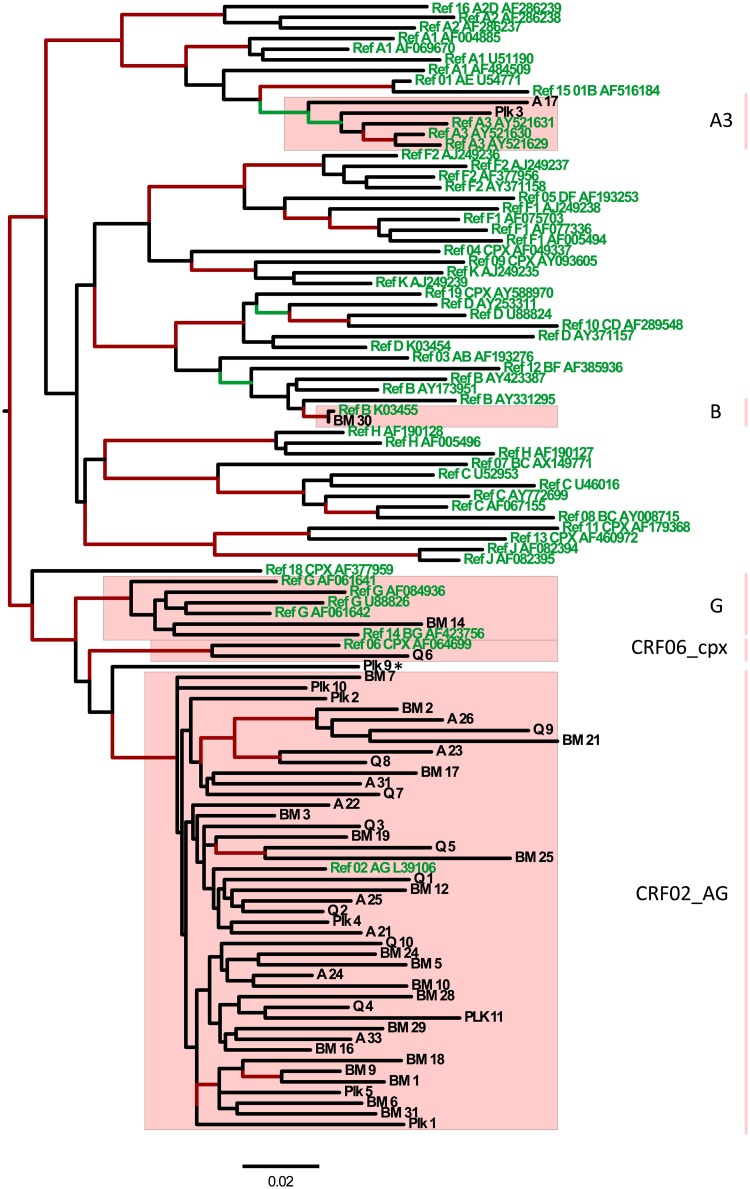
Maximum likelihood phylogenetic analysis of HIV-1 *pol* sequences (specimen names are indicated at tips in black font) from 48 women residing in Bissau, together with HIV-1 reference sequences (in green font), showed that sequences of the study participants belonged to five different subtypes/CRFs (highlighted within a box shaded pink, where the subtype/CRF names are shown to the right). One sequence (Plk_9) represented a putative recombinant between CRF06_cpx and CRF02_AG (indicated by an asterisk). Red internal branches indicate highly supported branches (aLRT-SH ≥0.90) while green internal branches indicate well-supported branches (0.87 ≤ aLRT-SH <0.90). The prefix of the taxa names indicates the collection site (BM, Bairro Militar Health Centre; A, Antula Health Centre; Q, Quelele Health Centre; and Plk, Plack-II Health Centre). The length of the horizontal bar below the phylogenetic tree corresponds to 0.02 nucleotide substitutions/site.

Phylogenetic subtyping and verification as described in the methods section identified that the vast majority of study participants (88%; 42 of 48) carried a HIV-1 *CRF02_AG* polymerase sequence, while *sub-subtype A3* (n = 2), *subtype G* (n = 1), *B* (n = 1) and *CRF06_cpx* (n = 1) were found less frequently. One sequence represented a putative recombinant *CRF06_xpx/CRF02_AG pol* sequence. There were no signs of phylogenetic clustering due to recent transmissions or laboratory contamination, since there were no clusters containing sequences with >95% nucleotide sequence similarity (Fig 1).

### Resistance mutations

Five individuals (5 of 48; 10.4%) carried major DRM. Four carried mutations towards NNRTI only (*K103N* and *K103N/S*) and one carried mutations to both NNRTI (*G190S*, *K101E*) and NRTI (*M184V*) No DRM toward protease inhibitors (PIs) was found. This represents a prevalence of DRM to NNRTI, NRTI and PI of 10.4% (95% CI: 4.5–22.2%), 2.1% (95% CI: 0.4–10.9%) and 0% (95% CI: 0.0–7.4%) respectively, corresponding to low levels of DRM to NRTI and PI and moderate levels of DRM to NNRTI. The individuals who presented with pretreatment DR are shown in Table 2. Women ≥25 years were more likely to harbour DRM (p = 0.049, FET; Table 3). Number of previous pregnancies, marital status, education, CD4 absolute count, CD4% and subtype/CRF were not associated with DRMs (Table 3).

**Table 2:** Study participants with pretreatment drug resistance.

Study Code	Age (years)	Marital Status	Ethnic Group	Previous Pregnancy	CD4^+^ T-cells (cells/ml)	CD4^+^ T-cells (%)	HIV-1 subtype/CRF	Mutation
BM14	25	Married	Fula	Yes	621	43.55	G	K103N
PLK5	36	Single	Balanta	Yes	417	16.97	CRF02_AG	K103N
BM28	26	Married	Mandinga	Yes	340	24.85	CRF02_AG	K103NS
A26	30	Married	Balanta	Yes	614	36.45	CRF02_AG	K103N
PLK10	30	Married	Felupe	No	356	23.14	CRF02_AG	K101E, G190S M184V

**K103N** and **K103N/S** cause intermediate to high-level resistance to the NNRTIs Nevirapine (NVP) and Efavirenz (EFV). **K101E** reduces susceptibility to NNRTIs NVP by 3 to 10-fold, to EFV by 1 to 5-fold, and to Etravirine (ETR) and Rilpivirine (RPV) by about 2-fold. **G190S** causes >50-fold decreased susceptibility to NNRTIs NVP and EFV. **M184V** reduce susceptibility to the NRTIs Lamivudine (3TC) and Emtricitabine (FTC) by >100-fold and also cause low-level resistance to Abacavir (ABC) and Didanosine (ddI).

**Table 3:** Medical and socioeconomic data of study participants in relation to pretreatment drug resistance (PDR).

Variable	No Total (n PDR)	p-value^1^
**Age (years)**		
15–24	22 (0)	
≥25	22 (5)	0.049
NA	4 (0)	
**Previous pregnancy**		
Yes	22 (4)	
No	23 (1)	0.187
NA	3 (0)	
**CD4 T-cell count (cells/ml)**		
>415	23 (3)	
≤415	23 (2)	1.00
NA	2 (0)	
**CD4T-cell %**		
>19.38	23 (4)	
≤19.38	21 (1)	0.176
NA	4 (0)	
**Education (years)**		
>3	19 (0)	
≤3	17 (4)	0.106
NA	12 (1)	
**Marital status**		
Married	32 (4)	
Not married	10 (1)	1.00
NA	6 (0)	

^1^ Two-tailed Fisher´s exact test

## Discussion

In this study we found 10.4% pretreatment DR in an ART-naïve group of pregnant women in Guinea Bissau. This is the first study of pretreatment HIV-1 DR in the country and the results raise some concerns. First line treatment in Guinea-Bissau, as in most LMIC relies on two NRTI and one NNRTI. In Guinea Bissau, the most commonly used NNRTI is Efavirenz [37] which is less effective in patients with the mutation *K103N/S*. The *K103N/S* mutation/s was found most frequently in this study and represents one of the most commonly detected pretreatment DR mutations in most LMIC since ART roll out [38].

We confirm our previous observations that *CRF02_AG* is the most common HIV form in Guinea-Bissau. In fact, our observation indicated an increased dominance of *CRF02_AG* in the country, from 57% in 1993–2008 to 88% in this study [13]. Among the specimens sequenced, we found no evidence of phylogenetic clustering, indicating that the study participants were not epidemiologically linked due to recent transmissions. However, further studies are necessary to confirm trends in prevalence of HIV subtypes/CRF in Guinea Bissau and association of clustering, if any. Moreover, the PCR protocol employed in our study was highly successful with a 100% amplification rate, indicating that the PCR protocol is useful for amplifying different HIV-1 subtypes/CRFs (21).

Previous studies in West Africa have found low levels of pretreatment DR in Guinea, moderate levels in Mali and levels varying from low to moderate in Cape Verde [39–43]. Levels of pretreatment DR in pregnant women were low in studies from Ghana and Nigeria, while moderate levels were observed in Burkina Faso [44–46]. Our study, which was performed twelve years after ART roll out in Guinea Bissau, found levels of pretreatment DR higher than the above mentioned studies, a difference that may be associated with the time of the studies after national ART scale up in the countries [8]. Similar observations have been made in other sub-Saharan countries after national ART scale-up and are mainly attributable to an increase in NNRTI resistance [38]. The specific mutations found in this study have also been found among patients failing treatment in Bissau, suggesting a link between acquired and transmitted drug resistance in the country [4, 47, 48].

WHO recommends performing threshold surveys in sites where a large proportion of individuals are likely to be young or recently infected and for these reasons the choice of antenatal clinics are representative investigation sites [19]. Several limitations are however evident in our study. Despite obtaining information of previous ART use and HIV diagnoses, almost half of the participants had previous pregnancies. We found no association between the number of previous pregnancies, marital status, education, CD4 absolute count, CD4% and subtype/CRF with DRMs. However, since the number of participants in the study was low, larger studies are needed to address these associations more accurately. It is possible that some participants concealed information regarding knowledge of HIV status and previous ART/PMTCT use which opens up for the possibility that a detected DR in an individual could be acquired and not transmitted by nature. Although we included participants from different clinics, they were all located in the capital of Bissau which may be less representative in the country as a whole due to regional differences in pretreatment DR [49]. There were also a number of challenges in recruiting participants at antenatal clinics in Bissau due to periods of stock-outs of HIV-antibody tests, limiting a full assessment of resistance in the region. In addition, acute and recent infections were not evaluated which may have led to an underestimation of the pretreatment DR level. Moreover, we included participants who were older than 25 year, while a 25 year age limit is recommended by the WHO. The purpose of this criterion is to limit the number of years of potential exposure to HIV transmission. However, and which has been discussed by Kasang et al. previously, it is questionable whether the age criterion is representative for a “sentinel group”, while the clinical reality is related to pre-existing HIVDR in all patients eligible for ART, irrespective of age and HIVDR origin (i.e. transmitted or acquired HIVDR) [50]. Regardless of whether the level of HIVDR reported here is predominately caused by transmission or selection due to undisclosed ART, it is likely to affect the first-line ART efficacy for a substantial number of individuals in the country.

In conclusion, even moderate levels of pretreatment DR in a presumably naïve population should raise concerns since it may represent a threat to the ART program in the country. As more individuals will start ART, more durable first line regimens are required. To respond to the threat of NNRTI drug resistance, the WHO recommends countries with a pre-treatment NNRTI resistance prevalence of 10% or higher to introduce a non–NNRTI-containing first-line regimen, for example the integrase inhibitor Dolutegravir [51]. Whether Guinea Bissau has reached this critical level should be further investigated. The results of the current study indicate that a substantial proportion of HIV-1 infected individuals are at risk for a suboptimal treatment compromising health and increasing the risk of transmission of drug resistant virus. Our findings provide novel data that may inform the development of more effective public health strategies in HIV prevention in Guinea-Bissau.
